# Role for extracellular vesicles in the tumour microenvironment

**DOI:** 10.1098/rstb.2016.0488

**Published:** 2017-11-20

**Authors:** Ana O'Loghlen

**Affiliations:** Epigenetics and Cellular Senescence Group, Blizard Institute, Barts and The London School of Medicine and Dentistry, Queen Mary University of London, 4 Newark Street, London E1 2AT, UK

**Keywords:** extracellular vesicles, EV, cancer, intercellular communication, microenvironment

## Abstract

Extracellular vesicles (EVs) are small-membrane vesicles secreted by most cells types with the role to provide intercellular communication both locally and systemically. The transfer of their content between cells, which includes nucleic acids, proteins and lipids, confers the means for these interactions and induces significant cellular behaviour changes in the receiving cell. EVs are implicated in the regulation of numerous physiological and pathological processes, including development and neurological and cardiovascular diseases. Importantly, it has been shown that EV signalling is essential in almost all the steps necessary for the progress of carcinomas, from primary tumours to metastasis. In this review, we will focus on the latest findings for EV biology in relation to cancer progression and the tumour microenvironment.

This article is part of the discussion meeting issue ‘Extracellular vesicles and the tumour microenvironment’.

## 1. Introduction

Extracellular vesicles (EVs) are a means of intercellular communication between neighbouring and distant cells. They contain nucleic acids, proteins and lipids, which can direct the fate of the recipient cell. EVs have been described to have a role in both physiological and pathological conditions and can modulate a number of cellular processes such as proliferation, migration, invasiveness and extracellular matrix (ECM) remodelling, generating a great interest in many different biological contexts [1].

From the 1960s, a number of groups observed that vesicles secreted by different cells in culture functional. While platelet-secreted vesicles regulated blood coagulation [2], it was found that EVs could transport trophic substances or nutrients to other cells [3]. Furthermore, different groups observed a role for secretory vesicles in reticulocyte maturation through recycling of transferrin and its receptor [4,5]. However, it was not until the late 1990s that a couple of studies found that immune cell-derived EVs could act as antigen presenters and T cell stimulators by expressing MHC class I and MHC class II molecules on their surface [6,7]. These studies presented for the first time an unconventional mechanism for intercellular communication, revealing the importance of a role for EVs in the immune system. Nowadays, it is widely recognized that EVs can have multiple functions in other physiological and pathological scenarios such as in cancer and in cardiovascular and neurodegenerative diseases [8].

## 2. Extracellular vesicles: biogenesis

**Figure 1.**
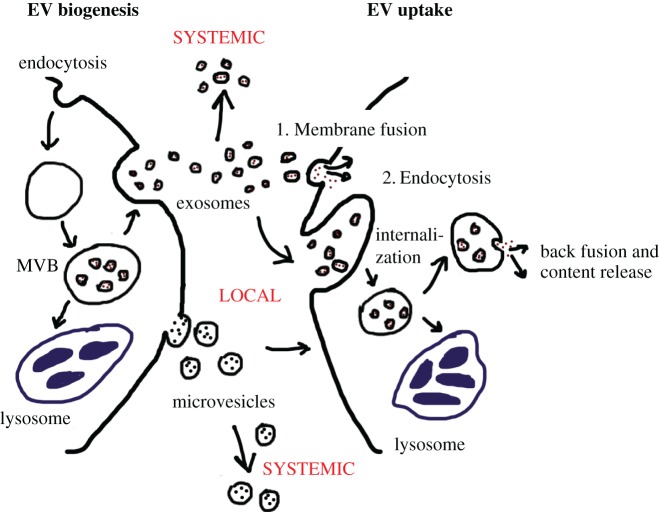
EV biogenesis and uptake. A simplified version of EV biogenesis is depicted on the left panel and EV uptake on the right. EVs can act locally, by affecting the behaviour of nearby cells or systemically, by travelling via blood or the lymphoid system and influencing cells long-distance. On the left panel, the plasma membrane of a cell can be endocytosed and trafficked to early endosomes and later to the multivesicular body (MVB). The MVB formed can either follow a degradation pathway fusing with lysosomes (blue) or proceed to release exosomes (small circles with red dots) to the extracellular space by fusing with the plasma membrane by exocytosis. On the other hand, microvesicles (big circles with black dots) are formed by direct shedding of the plasma membrane and release to the extracellular space. The right panel shows different possible routes for EV uptake. On one hand, EVs can establish specific binding with the plasma membrane followed by direct fusion of EV and cell membrane inducing the release of the EV cargo into the cytoplasm of the target cell (1). Altogether, various EVs can also be internalized by endocytosis, which once in the cytosol can either be directed to the lysosome for degradation or release their content to the cytosol by back-fusion of the EV membrane with the endosomal membrane (2).

EVs are lipid bilayer vesicles secreted to the extracellular space by cells. Their double membrane layer allows the EV content to be prevented from degradation from exogenous nucleases and proteases, facilitating long-term and long-distance transport of their cargo. Therefore, EVs allow cells to expand their intracellular transcriptome, proteome and lipidome to the extracellular space they reach, communicating their ‘status’ to other cells. EVs can be subdivided into three main categories depending on their subcellular origin: exosomes, microvesicles and apoptotic bodies [9,10]. Exosomes are the smallest of EVs, with a size ranging from 30–150 nm. They are generated inside multivesicular bodies (MVBs) and are released upon the fusion of the MVB with the plasma membrane [11]. Microvesicles have been previously referred to as ectosomes or oncosomes and range from 100 to 1000 nm. They are formed, matured and released by shedding from the plasma membrane of the cell (figure 1; left panel). Both microvesicles and exosomes comprise the accumulation of intracytosolic components although the protein and lipid composition between both EV subtypes differs [8,12]. Apoptotic bodies are the largest of all EVs (up to 5000 nm) and are released as membrane blebs of cells undergoing apoptosis. Throughout this review we will focus exclusively on the role of exosomes and microvesicles in the tumour microenvironment using the generic name of EVs, without specifying which type of EVs the original research studies are referring to.

### (a) Extracellular vesicle function in the extracellular space: uptake mechanisms

Upon the release by the parental cell, EVs can act either locally or systemically on neighbouring cells (figure 1). In fact, EVs can travel through blood and/or lymphoid nodes from other tissues [13]. It is therefore not surprising that EVs have been found in a number of biological fluids including plasma, urine, breast milk, semen, cerebrospinal fluid and saliva [8].

The exact EV uptake mechanisms for recipient cells are not completely understood but different groups have provided evidence for EV cargo uptake by either: (i) direct EV fusion at the plasma membrane, releasing the EV cytosol content into the cytoplasmic compartment of the target cells; or (ii) by uptake through endocytosis followed by back-fusion of the EV with the endosomal membrane releasing their contents to the cytoplasm of the recipient cell (figure 1) [8]. In fact, in 2007, Valadi *et al.* demonstrated that mRNA and microRNA (miR) could be transferred via EVs from mouse to human mast cells. They also showed that the RNA content was functional as they found mouse proteins in the human recipient cells [14]. The delivery of EVs derived from dendritic cells (DC) loaded with an siRNA targeting GAPDH showed a reduction in the expression levels of GAPDH in neurons, microglia, oligodendrocytes demonstrated that the siRNA was effectively transferred and functional [15]. Furthermore, performing membrane fusion assays using EVs loaded with luciferin substrate to treat luciferase-expressing cells resulted in production of bioluminescence in the recipient cells [16]. It was also demonstrated that heparan sulphate proteoglycans (HSPGs) function as essential receptors for the endocytosis of cancer-derived EV [17] and recently, Neuropilin-1 has been confirmed as a receptor for extracellular miRNA and AGO2/miRNA complexes internalization in recipient cells [18]. Altogether, these and many other studies have shown that EVs can be effectively taken up by recipient cells, although it is possible that the EV uptake mechanism is cell-type– and context-dependent.

EVs do seem to have some characteristics that favour cell-specific uptake. For example, EVs derived from platelets preferentially transferred tissue factor (TF) to macrophages but not neutrophils [8], while EVs derived from different tumours are taken up by cells within their preferential metastatic site and depend on their preferred integrin expressed [19,20] and exosomes derived from K562 or MT4 cells were internalized more efficiently by phagocytes than by non-phagocytic cells [21]. These heterogeneous responses are not surprising though the particular proteins involved from both EVs and recipient cells remain to be elucidated.

## 3. Role for extracellular vesicles in cancer

The complexity of tumours is becoming increasingly recognized with the view of tumours formed exclusively from cancer cells now being obsolete. In fact, a variety of cell types such as fibroblasts, lymphocytes, inflammatory cells, epithelial cells, endothelial cells and mesenchymal stem cells can be found within the tumour microenvironment [22]. Although for years the main mediator for the tumour intercellular communication was attributed to secreted proteins like growth factors, cytokines and chemokines recent advances in cancer biology show that EVs play a key role in this communication process [8]. Therefore, the need for a coordinated multistep programme and a multifaceted signalling network between all the different cell types is necessary for the success of tumour development [22].

### (a) Extracellular vesicles released by tumour cells can both suppress and activate the immune system

EVs have been shown to be involved in the regulation of an immune response and therefore much attention has been brought in the cancer field to the interplay between tumour EVs and the immune system regulation [23]. Importantly, it seems that the initial local interaction between tumour cells and the innate immune response might be critical in influencing tumour fate [19].

Tumour-derived EVs are a reflection of the protein composition of the parental cell. Therefore, EVs can contain tumour-specific antigens such as carcinoembryonic antigen (CEA) and mesothelin [24]. As a consequence, tumour-specific antigens can induce the maturation of antigen-presenting cells (APC), stimulating cytotoxic CD8^+^ T and natural killer (NK) cells, eventually eliminating cancer cells [25,26]. This anti-tumour response is in line with previous reports, where EVs derived from DC cells functionally express MHC Class I and II molecules, inducing anti-tumour responses dependent on CD8^+^ T lymphocyte activation [7,27]. Interestingly, Headly *et al.* have shown that circulating tumour cells from the lung release EVs that migrate along the lung vasculature and are subsequently taken up by myeloid cells. As a consequence, this activates DC cells that initiate an anti-tumour response [28]. Another recent study has also shown that the loss of the Hippo pathway kinases large tumour suppressor 1 and 2 (LATS1/2) in tumour cells inhibits tumour growth by nucleic-acid-rich-EVs, which induce a type I interferon response (IFN) via the Toll-like receptors-MYD88/TRIF pathway [29].

Although the activation of the immune system can initially reduce tumour growth, cancer cells generally have defence mechanisms to evade immune surveillance. Pucci *et al*. have found that tumour-derived EVs preferentially bind subcapsular sinus of lymph nodes, where a specialized population of macrophages (CD169^+^) block the dissemination of cancer EVs. Interestingly, this barrier is altered during cancer allowing the tumour-derived EVs to travel along the lymph nodes and activate B lymphocytes promoting tumour growth [30].

In fact, tumour-derived EVs create an immunosuppressive niche that protects the tumour from the immune system [31,32]. For example, EVs derived from breast cancer cells were shown to activate tumour-activated macrophages (TAMs), inducing the secretion of IL-6, tumour necrosis factor alpha (TNFα), granulocyte-colony stimulating factor (G-CSF) and CCL2 by NK-κB activation and promoting vascularization and angiogenesis [33]. Furthermore, TGFβ was found to be essential for the recruitment of tumour-associated neutrophils to the tumour [34], and breast cancer–derived EVs can immobilize neutrophils in the tumour promoting cancer progression [35]. In addition, EVs derived from serum of patients with cancer have been shown to express FasL and TRAIL as transmembrane proteins, activating programmed cell death or apoptosis in cytotoxic CD8^+^ T cells [31]. The TNF superfamily member, CD95 L, is also found in tumour EVs and mediates immune evasion, and the presence of CD11b in tumour EVs suppresses antigen-specific responses via an MHC class II–dependent mechanism [24]. The existence of this immunosuppressive niche is reinforced by the activation of DC by tumour-derived EVs [24,32], and by favouring the generation of myeloid-derived suppressor cells (MDSCs), that contain prostaglandin E2 (PGE2), transforming growth factor-β (TGFβ) and heat shock protein 72 (HSP72) in their secreted vesicles [24]. All these studies show that EVs derived from tumour cells present a wide range of antigens capable of evading immune surveillance.

### (b) Tumour-derived extracellular vesicles influence the transition to metastasis

Apart from evading immune surveillance, cancer cells need alternative pathways in order to successfully grow and colonize foreign tissues. In this section, we will explain how tumour-derived EVs contribute to the step necessary to transition to metastasis such as inducing changes in the tumour stroma, promoting angiogenesis and favouring epithelial–mesenchymal transition (EMT).

**Figure 2.**
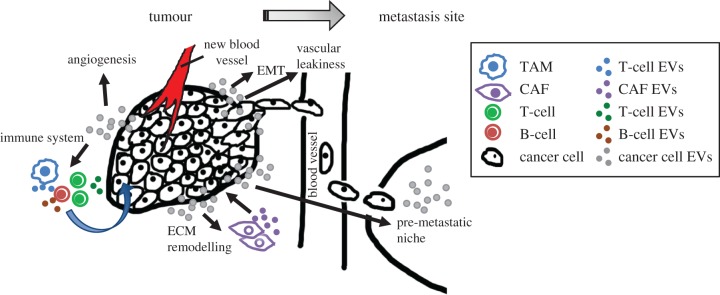
Tumour-derived EVs influence the microenvironment to promote tumour progression. The tumour microenvironment is comprised of a variety of cell types, which interact with each other via different signalling mechanisms. Tumour-derived EVs induce the activation of tissue-resident fibroblasts into myofibroblasts, cancer-associated fibroblasts (CAFs), which in turn modify the ECM favouring cancer cell growth and the recruitment of immune cells. In fact, EVs derived from cancer cells can exert both anti-tumour and pro-tumour activities on different cells of the immune system, which as a consequence secrete their own EVs altering the behaviour of cancer cells. Furthermore, tumour-derived EVs contribute to numerous steps required for the progression from a primary tumour to the final stages of metastasis, such as stimulating epithelial to mesenchymal transition (EMT), the formation of new blood vessels (angiogenesis), vascular leakiness and pre-conditioning of the premetastatic niche for ‘foreign cell’ establishment.

For the tumour to continue progressing, a complex stromal support is needed [22]. Cancer-stroma is mainly composed of the cancer-associated fibroblasts (CAFs) subtype myofibroblasts, which release enzyme-degrading proteases or metalloproteases (MMPs) that contribute to the formation of desmoplastic stroma, a feature of advanced carcinomas. EVs containing TGFβ have been shown to drive tissue-resident fibroblasts into myofibroblasts demonstrated by the expression of α-smooth muscle actin (α-SMA) [36,37]. Furthermore, CAFs can secrete EVs inducing protrusive activity and mobility in breast cancer cells by a Wnt-driven planar cell polarity [38] and transfer radiation and chemotherapy resistance in the form of EV messaging [39]. In addition, a recent paper has shown that melanoma cells release EVs carrying miR-211, inducing the activation of CAFs in the dermal stroma [40]. Therefore, the communication between CAFs and cancer cells via EVs is an important intercellular communication mechanism that induces changes in the tumour microenvironment (figure 2).

EVs derived from several human cancer cells have been shown to induce angiogenesis. One particular example is EVs derived from renal cancer cells that promote angiogenesis in the lung [41]. In addition, epidermal growth factor receptor, EGFR-enriched EVs produced by cancer cells are uptaken by endothelial cells, inducing vascular endothelial growth factor (VEGF) and VEGF receptor 2 expression [42], while TF-containing EVs upregulate angiogenesis [43]. Furthermore, the transfer of the microRNA *miR-150* from EVs to TAM leads to a proangiogenic environment through the secretion of VEGF [44].

Tumour-derived EVs also can activate EMT transition in epithelial cells triggering their loss of cell adhesion. The loss of adhesion alters the encapsulated structure of the primary tumour facilitating the release of tumour cells to distant sites to induce metastasis. In fact, a number of studies have observed that EVs derived from Madine-Darby canine kidney epithelial cells and the breast metastatic cell line MDA-MB-231 can induce EMT in recipient cells [45,46].

Overall, all these series of events allow the tumour to progress and metastasize.

### (c) Extracellular vesicles play an important role in the formation of secondary tumours and metastasis

As tumours evolve, their intercellular communication becomes distorted, with EVs derived from tumour cells affecting all hallmarks of cancer [22].

In 1889 Stephen Paget observed that different tumour types have preferential metastatic sites [47], while Ernest Fuchs perceived that those sites must be predisposed for allowing ‘foreign cell’ growth. In fact, the role for EVs in creating an ideal premetastatic niche is becoming increasingly recognized. The contribution to soluble factors and EVs from a subtype of pancreatic cancer cells to predispose the lymphoid node and lung was first made by Jung *et al.* [48]. However, many other studies followed this observation.

The blood vessels produced within tumours are typically aberrant allowing vascular leakiness and abnormal endothelial cells morphology [22]. Tumour-derived EVs also contribute to the induction of vascular leakiness, an additional factor that contributes to EV-promoted metastasis. Melanoma-derived EVs induce the upregulation of S100 proteins and TNFα, causing vascular leakiness, inflammation and bone marrow progenitor recruitment [49]. Similarly, breast-derived EVs also promote vascular leakiness by activating Src kinase signalling pathway [50] and through the release of exosomal *miR-105*, which targets the mRNA encoding the tight junction protein ZO-1 in endothelial cells [51].

Several groups have found that upregulation of S100 and MMP proteins mediated by tumour EVs creates a premetastatic niche, either by enhancing vascular leakiness [49] or by TLR3 activation [52]. In fact, MMPs present in tumour-derived EVs can influence the ECM, inducing morphological changes ultimately leading to metastasis [38,53]. Interestingly, a recent study has found a specific pattern for integrin expression in tumour-derived EVs, which directs EVs to specific metastatic sites via S100 upregulation. Thus, EVs containing integrins α6β4 and α6β1 have been associated with lung metastasis, while αvβ5-expressing EVs are linked to liver metastasis [50]. Another study found that fibronectin (FN)-integrin α5β1 EVs derived from fibrosarcoma promoted cell migration *in vitro* and *in vivo* [54]. In fact, integrin signalling via focal adhesion kinase (FAK) is considered a possible mechanism of EV signalling in cancer [55].

In recent years, the transfer of RNA within tumour EVs has generated a great interest in the cancer community. A study by the Lötvall group showed that mast cell–derived EVs contain and transfer miR and mRNA to recipient cells, therefore regulating gene expression [14]. Likewise, several miR targeting the tumour suppressor gene PTEN have been found in astrocyte-derived EVs, enhancing the growth of brain metastatic cells [56]. Interestingly, exosome cargo can also influence glucose metabolism. A recent study showed that breast-derived *mir-122* can be transferred to stromal fibroblasts and prevent glucose uptake by downregulating pyruvate kinase [57]. However, further thorough investigation is needed to conclusively confirm EV-dependent transport and expression of miR.

Once the premetastatic niche is conditioned by tumour EVs, this influences the recruitment of additional cells to promote the growth of secondary tumours. Both melanoma and pancreatic tumour EVs induce the recruitment of bone marrow-derived cells (BMDC) [49,58]. In particular, EVs from pancreatic cancer cells activate resident macrophages in the liver (Kupffer cells), which release TGFβ. TGFβ in turn activates hepatic stellate cells (HSCs) that induce ECM remodelling prompting the recruitment of BMDC [58]. It seems that the receptor tyrosine kinase receptor Met plays a key role in metastatic EV-mediated preconditioning. Met can be found in tumour EVs and can be transferred to recipient cells, which in turn promote tumorigenesis [49,59,60]. However, cancer cells also release EVs containing other oncogenic proteins. The oncogene KIT was found in EVs derived from gastrointestinal tumours [61], while a truncated oncogenic form of the epidermal growth factor receptor (EGFRvIII) can be uptaken by cells negative for the receptor via EV transfer [62]. Altogether, these studies suggest that the presence of the oncogene Met in EVs is not the only mechanism involved in promoting metastasis.

## 4. Future directions

Tumour-derived EVs prepare the premetastatic niche for metastasis. To deal with this issue, a recent study created an artificial premetastatic niche by embedding tumour-derived EVs in a 3D scaffold device, which they called M-Trap. By implanting M-Trap in an animal model, they could observe a reduction in the metastatic potential of ovarian tumour cells and an increase in the survival rate of the mice [63]. Altogether this technique could potentially present a promising approach to deal with cancer metastasis, although a more detailed investigation into different types of cancer and the precise mechanism implicated would be needed.

Another question that remains to be answered is: *Why not inhibit EV biogenesis if it is so detrimental in cancer?* Several studies have tried this approach with more or less success. Interference with some Ras-related RAB proteins, which are essential for EV biogenesis, has been shown to reduce migration, growth and metastasis [1]. However, although a reduction in the metastatic potential induced by injecting EVs from RAB-depleted cells was observed, metastasis was not completely abolished, suggesting either additional mechanisms unrelated to EV biogenesis are implicated or that affecting EV biogenesis influences other cellular signalling pathways. This further highlights the existing complexity of the different EV subtypes and their functionality, which is an area of enormous interest in the field. In fact, further basic and translational research on this topic is likely to pay dividends in terms of regaining control of our understanding of the cancer microenvironment and metastatic dissemination.

## Data accessibility

This article has no additional data.
